# Alloy formation during molecular beam epitaxy growth of Si-doped InAs nanowires on GaAs[111]B

**DOI:** 10.1107/S0021889813010522

**Published:** 2013-06-07

**Authors:** Anton Davydok, Torsten Rieger, Andreas Biermanns, Muhammad Saqib, Thomas Grap, Mihail Ion Lepsa, Ullrich Pietsch

**Affiliations:** a Festkörperphysik, Universität Siegen, Walter-Flex-Strasse 3, Siegen, Nordrhein Westfalen 57072, Germany; b Peter Grünberg Institut (PGI-9), Forschungzentrum Jülich, Jülich, Nordrhein Westfalen 52425, Germany; c Jülich Aachen Research Alliance for Fundamentals of Future Information Technology (JARA-FIT), Germany

**Keywords:** semiconductor nanowires, molecular beam epitaxy (MBE) growth, X-ray diffraction

## Abstract

Vertically aligned InAs nanowires (NWs) doped with Si were grown self-assisted by molecular beam epitaxy on GaAs[111]B substrates covered with a thin SiO*_x_* layer. Using out-of-plane X-ray diffraction, the influence of Si supply on the growth process and nanostructure formation was studied. It was found that the number of parasitic crystallites grown between the NWs increases with increasing Si flux. In addition, the formation of a Ga_0.2_In_0.8_As alloy was observed if the growth was performed on samples covered by a defective oxide layer. This alloy formation is observed within the crystallites and not within the nanowires. The Ga concentration is determined from the lattice mismatch of the crystallites relative to the InAs nanowires. No alloy formation is found for samples with faultless oxide layers.

## Abstract 2

An investigation of the influence of Si supply on the molecular beam epitaxy growth process and morphology of InAs nanowires grown on GaAs[111]B substrates is presented.

## 1. Introduction

Group III–V semiconductor nanowires (NWs) demonstrate interesting electrical and optical properties and are very promising for the fabrication of future semiconductor devices. For example, such NWs are already found in applications as tunnel diodes (Wallentin *et al.*, 2010 ▶), photo diode sensors (Wei *et al.*, 2009 ▶) and solar cells (Tang *et al.*, 2011 ▶). The most common way to realize the growth process of semiconductor nanowires is the vapour–liquid–solid mechanism (Wagner & Ellis, 1964 ▶), in which a metallic droplet acts as a seed for epitaxial NW growth. Here, the seed particle may be supplied either externally (*e.g.* gold) or by one of the NW constituents (*e.g.* Ga for GaAs, so-called self-assisted growth) (Bauer *et al.*, 2010 ▶). Using molecular beam epitaxy (MBE), NWs from almost any semiconductor material can be combined with nearly any substrate, independent of lattice mismatches (Schubert *et al.*, 2004 ▶). The additional use of a doping material can provide variability in device design and selectivity for future device structures (Li *et al.*, 2011 ▶). An important prerequisite for the self-assisted growth of the nanowires is the existence of a good quality oxide layer on the substrate regarding both homogeneity and thickness (Fontcuberta i Morral *et al.*, 2008 ▶; Krogstrup *et al.*, 2010 ▶). The NWs grow epitaxically on the underlying substrate through small pinholes within this oxide layer, which are either present from the beginning or created during the initial stage of growth. Consequently, no NWs are obtained if the oxide thickness exceeds a certain value (Mandl *et al.*, 2010 ▶). Among the different possibilities to obtain the thin oxide layer, one is to convert spin-coated hydrogen silsesquioxane (HSQ) into SiO_*x*_ by thermal treatment as introduced by Rieger *et al.* (2012 ▶). Recently, several studies have focused on the mechanism and the limitation of dopant incorporation into the nanowires (Stoica & Calarco, 2011 ▶, for example). In this sense, it was shown that Si doping systematically affects the morphology of MBE-grown III–nitride NWs. An increase in the doping concentration leads to a reduction of the NW density with an increase in the average NW diameter. The X-ray diffraction technique provides the possibility of NW characterization in terms of lattice parameters and structural composition (Davydok *et al.*, 2012 ▶). In the present paper, we use X-ray diffraction methods to study the growth of InAs nanowires on oxide-covered GaAs surfaces as a function of the initial substrate preparation and Si supply level for doping. We observe that a large number of parasitic crystallites form on the surface with increasing Si supply. In the case of growth on defective oxide layers, Ga is dissolved from the substrate and is alloyed to a Ga_0.2_In_0.8_As solid solution, which is preferentially found within the parasitic crystallites.

## 2. MBE growth

**Figure 1:**
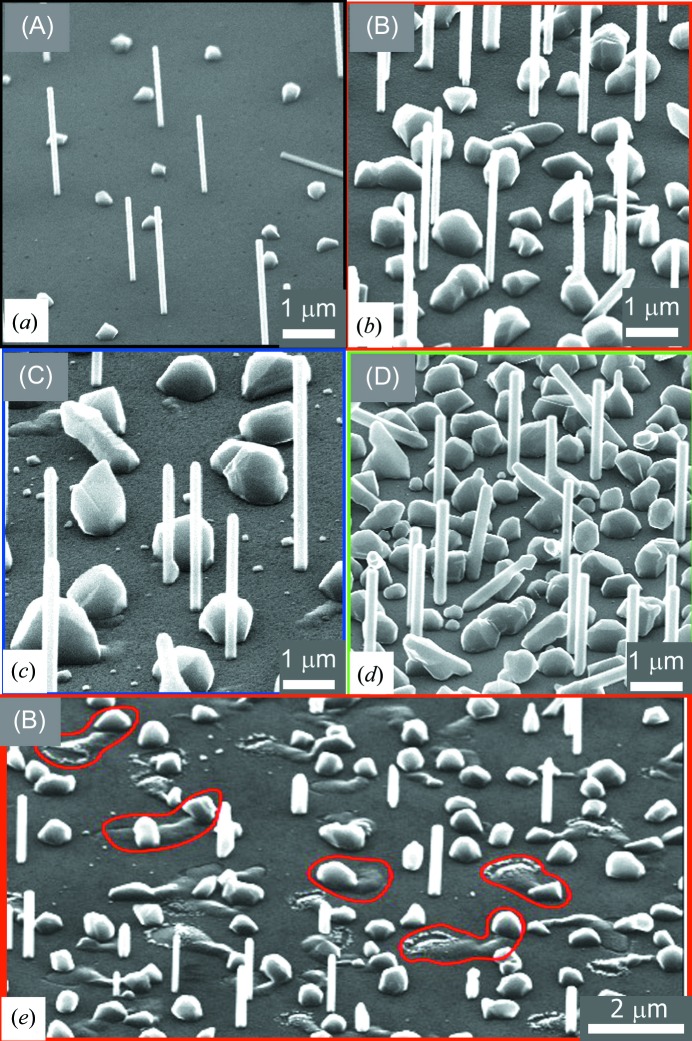
SEM images of (*a*) undoped sample (A) of series 1, (*b*) sample (B) with doping level 1 × 10^17^ cm^−3^ from series 1, (*c*) sample (C) with doping level 1 × 10^18^ cm^−3^ from series 1 and (*d*) sample (D) from series 2 with doping level 1 × 10^17^ cm^−3^. (*e*) Large area image of sample (B); places of damaged oxide surface are marked with red lines.

InAs NWs were grown on GaAs[111]B substrates, *i.e.* on substrates having As-terminated (111) crystal orientation (Rekaya *et al.*, 2005 ▶). Two sets of samples were grown in a Varian GEN-II-MBE machine at a substrate temperature of 803 K. Prior to the growth, the substrate was spin coated with HSQ diluted with methyl isobutyl ketone; there followed an annealing step at 573 K to convert the HSQ into SiO_*x*_. Then, the oxide layer was thinned by wet chemical etching in diluted HF. In the first sample series, a 12 nm-thick SiO_*x*_ layer was etched, finally giving a 6 nm-thick defective oxide layer with several large openings that can be observed in scanning electron microscopy (SEM) images (see Fig. 1 ▶ *e*). InAs NW growth was performed using a nominal planar In growth rate of 0.025 µm h^−1^ and an As_4_ partial pressure between 0.8 × 10^−6^ and 1.2 × 10^−6^ Torr (1 Torr = 133.32 Pa), leading to an NW growth rate of 0.3–0.4 µm h^−1^ at the growth temperature of 803 K. NWs were grown for 3–4 h (depending on the As_4_ partial pressure used), resulting in ∼1.2 µm-long NWs. During the growth, Si adatoms were supplied additionally in order to dope the NWs. The equivalent doping concentrations for planar layer growth were zero [undoped, sample (A)], 1 × 10^17^ cm^−3^ [sample (B)], 1 × 10^18^ cm^−3^ [sample (C)] and 5 × 10^18^ cm^−3^ (not shown). The substrates of the second series were prepared using a higher dilution of HSQ and without the etching procedure, resulting in a homogeneous oxide thickness of 6 nm. Similar to the first series, one undoped sample and samples with doping levels of 1 × 10^17^ cm^−3^ [sample (D)], 1 × 10^18^ cm^−3^ and 8 × 10^19^ cm^−3^ were also grown.

Prior to XRD measurements, all samples were inspected using SEM. Fig. 1 ▶ presents exemplary SEM images of the investigated samples. In Fig. 1 ▶(*a*), a sample grown without Si supply is shown. The NWs have a typical diameter of 100 nm and length of 1.2 µm. In addition, crystallites are found on the surface in between the NWs. This parasitic growth is commonly observed in self-assisted NW growth by MBE (Dimakis *et al.*, 2011 ▶). Figs. 1 ▶(*b*) and 1 ▶(*c*) show the samples (B) and (C) of the first series grown in the presence of silicon. Increasing the doping concentration, the surface coverage by parasitic crystallites increases. Simultaneously, the density of InAs NWs reduces with increasing doping level. This behaviour is independent of the substrate preparation mentioned above and it was observed also on the second series of samples. For comparison with samples grown on a defective oxide layer [samples (B) and (C)], Fig. 1 ▶(*d*) shows an Si-doped sample grown on a nondefective oxide layer [sample (D)]. Another view of sample (B) with a surface area in which the defective oxide layer has larger openings (encircled areas) is shown in Fig. 1 ▶(*e*). The inspection of several SEM images indicates that additional material emerges from these defective areas, usually forming a trace towards one of the parasitic crystallites. This will be further discussed below.

**Figure 2:**
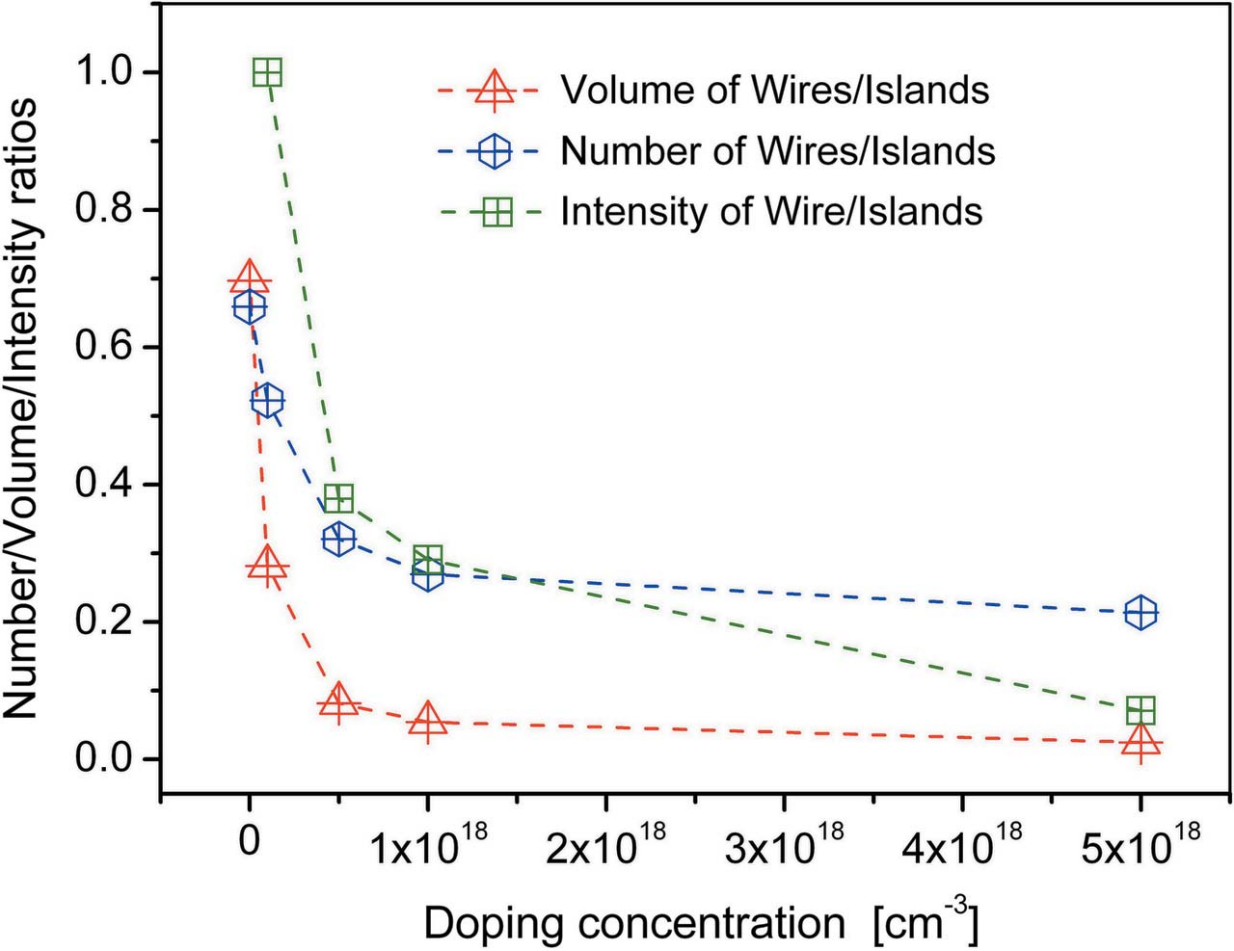
Ratios of NW volume/number/intensity to crystallite volume/number/intensity as a function of Si doping concentration.

For statistical analysis, the ratio between parasitic crystallite growth and nanowire growth has been determined from the SEM images taken for different doping concentrations of series 1. Fig. 2 ▶ presents the results of this analysis. The triangles show the number ratio of wires to crystallites (determined on a surface area of 20 × 10 µm). Already for the undoped sample, the determined ratio of 0.7 indicates that on average more crystallites than NWs are grown. With increasing doping level, this ratio quickly decreases down to a value of 0.25, showing that the growth of each nanowire is accompanied by the growth of four crystallites. In addition, one can extract from the SEM images that, on average, the size of the islands is larger in highly doped samples compared with the lower or undoped ones. This finding has been considered to count the volume ratio of NWs to crystallites. This volume ratio is shown by the blue hexagons in Fig. 2 ▶.

## 3. Experimental technique

X-ray diffraction experiments were performed in order to study the evolution of the lattice parameters and the crystal structure of the grown InAs nanowires. The experiments were performed at beamline BL9 of the DELTA synchrotron source in Dortmund, Germany. All measurements were done in a coplanar scattering geometry with the samples mounted horizontally. A parallel monochromatic X-ray beam of wavelength λ = 1.23 Å was obtained using a monochromator and a set of slits with the size 200 × 500 µm (vertical and horizontal size, respectively) in front of the sample, illuminating the surface with the incidence angle ω. The diffracted intensity was recorded using a two-dimensional detector (PILATUS) and integrated along the direction parallel to the sample surface, in order to mimic a one-dimensional detector measuring the diffracted intensity as a function of the scattering angle 2Θ°.

For comparison, the measured intensity distribution as a function of the angles ω–2Θ has been transferred into wavelength-independent reciprocal coordinates using the following expressions: *q_x_* = (2π/λ)[cos(2Θ − ω) − cos(ω)] and *q_z_* = (2π/λ)[sin(ω) + sin(2Θ − ω)]. Here, *q_z_* describes the momentum transfer along the surface normal and *q_x_* the momentum transfer parallel to the projection of the incoming X-ray beam on the surface.

For all samples, reciprocal space maps (RSMs) were recorded around the symmetric GaAs 111 reflection as well as around the asymmetric 331 one.

## 4. Results and discussion

**Figure 3:**
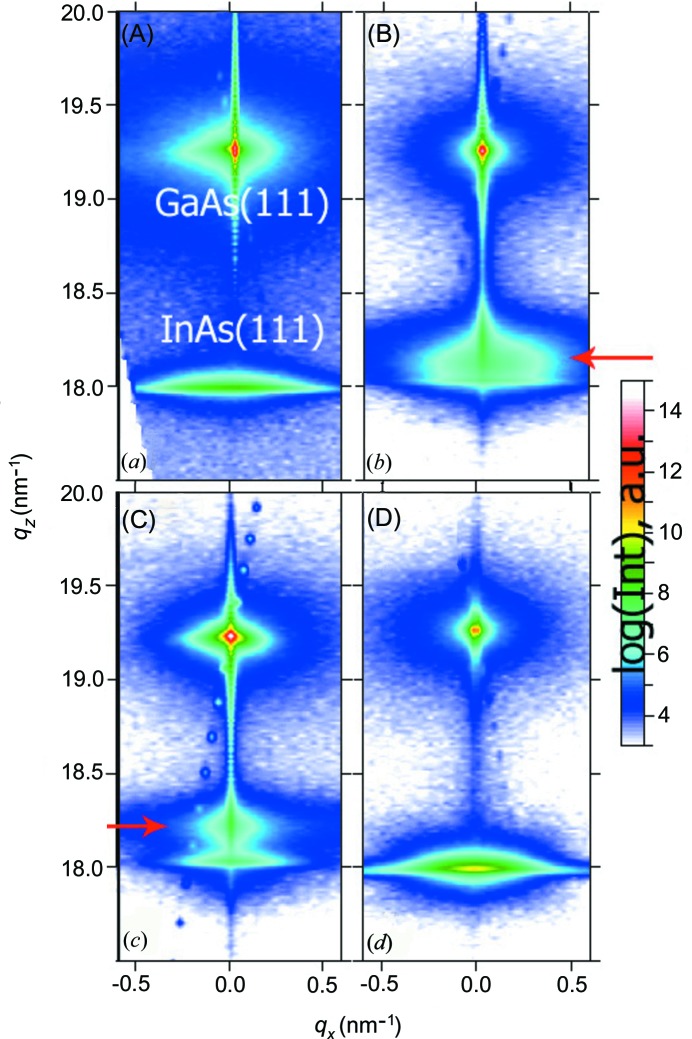
RSMs measured around InAs and GaAs 111 reflections on samples (A)–(C) grown on defective SiO*_x_* layers with increasing doping concentration: apart from the substrate and InAs reflections, an additional reflection appeared for the doped samples (red arrows). Sample (D) was grown on the ‘good’ surface.

Figs. 3 ▶(*a*)–3 ▶(*d*) show the measured reciprocal space maps around the symmetric 111 reflection for samples (A)–(D), respectively. The strong 111 Bragg reflection of the GaAs substrate, seen in the upper part of the maps at *q_z_* = 19.25 nm^−1^, has been used as reference on all the samples. For the undoped sample (A) (Fig. 3 ▶ *a*), a second peak located at *q_z_* = 17.98 nm^−1^ is seen, corresponding to the expected position of pure InAs with lattice constant *a*_InAs_ = 6.058 Å. Using X-ray reflection in the symmetric scattering geometry, one gets access to the atomic planes parallel to the surface. In this geometry, the full width at half-maximum (FWHM) is determined by the size and tilt of the scattering objects, and is not sensitive to the influence of planar defects. The InAs peak has an FWHM along the *q_x_* direction of 0.16 nm^−1^, which is larger than expected from the nanowire diameter (∼100 nm) (2π/100 nm ≃ 0.06 nm^−1^). The difference in width can be attributed to a small angular distribution of the orientation of different NWs with respect to the [111] axis of the substrate or to the contribution of parasitic crystallites which also are not perfectly aligned along the [111] direction.

The reciprocal space maps for the doped samples are shown in Figs. 3 ▶(*b*), 3 ▶(*c*) and 3 ▶(*d*). Note that the doped sample (D) taken from second series (Fig. 3 ▶ *d*) shows no difference from the diffraction pattern measured for the undoped sample (A) (Fig. 3 ▶ *a*). This holds for all samples of this series and indicates that the presence of Si does not lead to a change of crystal structure or lattice parameters in the NWs or crystallites, independent of doping concentration.

A different evolution is observed in the case of growth on the defective oxide layer. For sample (B), a second peak emerges, being located at a significantly larger vertical momentum transfer of *q*_*z*_ = 18.20 nm^−1^ (marked by an arrow in Fig. 3 ▶ *b*). Compared with the initial InAs signal, this peak has a larger FWHM along the *q*_*z*_ direction, but a narrower distribution along *q*_*x*_, with FWHM Δ*q*_*x*_ = 0.08 nm^−1^. With the increase of the doping level up to 1 × 10^18^ cm^−3^ – sample (C) – this additional reflection becomes more intense compared with the InAs peak and exhibits smaller FWHMs of Δ*q*_*x*_ = 0.02 nm^−1^ and Δ*q*_*z*_ = 0.15 nm^−1^ along the horizontal and vertical directions, respectively. Because the width of a Bragg peak is inversely proportional to the size of the diffracting objects, this indicates that these peaks are arising from laterally larger, but vertically smaller, objects compared with the NWs, with typical size of (2π)/Δ*q*_*x*_ ≃ 300 nm diameter and (2π)/Δ*q*_*z*_ ≃ 40 nm height, respectively.

Correlated with the SEM images shown in Fig. 1 ▶, our finding suggests that this diffraction signal might be originating from the crystallites growing in between the NWs, dominating the surface coverage for higher doping concentration. Assuming that the crystal structure of the material causing this Bragg reflection is a cubic zinc-blende one, the peak position corresponds to a lattice parameter of 5.984 Å, being 1.2% smaller than that of InAs.

**Figure 4:**
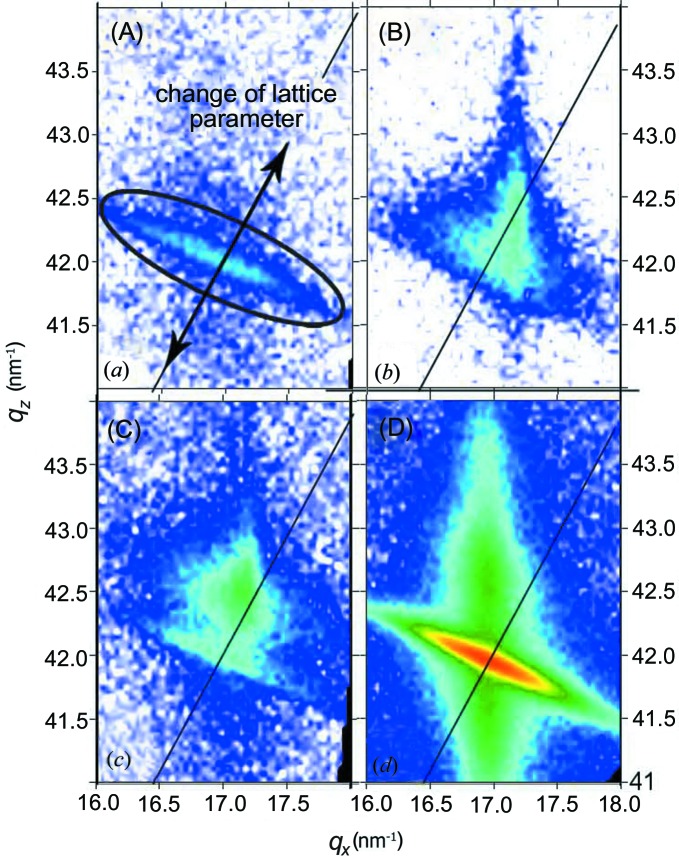
RSMs of the InAs 331 reflection from samples (A)–(D): the inclination of the NW reflection indicates the angular distribution of NW orientation; the vertical strikes observed independent of doping are coming from crystallites; in the case of samples grown on etched SiO_*x*_ layers and with Si doping, the slight shift of the additional peak along the diagonal (black) line indicates a structure with different lattice parameter from InAs.

In order to verify this result, we determined the crystal structure of the new feature by measurements in asymmetric diffraction geometry around the InAs 331 reflection. Fig. 4 ▶ shows the reciprocal space maps in the *q*_*x*_ *q*_*z*_ plane for the samples discussed above.

For samples (A) and (D), the reciprocal space maps show an elongated Bragg reflection, indicated by the ellipse drawn in Fig. 4 ▶(*a*). This reflection is caused by the grown InAs NWs, and the angular tilt of the nanowires will spread the Bragg peak along a circle of constant |*q*|. In the case of sample (D), a strong diffuse scattering signal is observed along the *q*_*z*_ direction. This can be explained by the existence of stacking faults within the grown InAs nanowires as has been reported by Kriegner *et al.* (2011 ▶). Indeed, exemplary transmission electron microscopy measurements on selected nanowires show that the crystal structure of the NWs is cubic zinc-blende with a high density of rotational twins (Blömers *et al.*, 2011 ▶) (not shown). This streak is observed for all the samples from series 2, independently of the doping level, and the samples grown without the presence of Si in both series 1 and 2 (not shown). Owing to the reduced overall intensity measured on sample (A), as a result of a lower overall surface coverage with NWs and crystallites on this sample, this streak is hardly visible here. However, the general shapes of the Bragg reflections are similar for all cases. A different picture is observed for sample (B). The initial Bragg peak is superimposed by a laterally sharper, but vertically more elongated, feature. As seen, this peak is displaced along the radial direction in reciprocal space, indicated by the black line in the figures. This displacement indicates that the additional Bragg peak corresponds to material grown in a cubic phase and cannot be explained by strained InAs, because the tetragonal distortion of the unit cells in strained InAs would require a lateral compression (larger *q*_*x*_) together with a vertical expansion (smaller *q*_*z*_), which is not observed here.

In the case of sample (C) with higher doping level, this additional reflection is more pronounced in intensity, but otherwise maintains the same position. The most likely explanation of the observed lattice parameter change is an alloy formation between the supplied material (InAs) and one component of the substrate (Ga). Based on the determined lattice parameter of 5.984 Å and using Vegard’s law, a Ga_0.2_In_0.8_As alloy might form in the presence of Si on the defective oxide layer. We note that the additional material cannot be explained by the additional silicon, as Si itself has a smaller lattice parameter compared with GaAs and the amount of supplied silicon is too small (doping level) to explain the appearance of such islands.

The shape of the additional Bragg reflection indicates that the corresponding material has typical dimensions of 300 nm in diameter and 40 nm in height (see above), and hence this reflection is presumably caused by material grown in the parasitic crystallites. To support this assumption, we determined the integrated intensities of the Bragg reflection attributed to the NWs and the additional Bragg reflection present in series 1. The ratio of these intensities, which is proportional to the scattering volume of the respective phases, is shown by open squares in Fig. 2 ▶. Owing to the missing island signal on the undoped sample the respective ratio is infinite and was set to unity in Fig. 2 ▶. The intensity ratio shows the same tendency as obtained from the analysis of the SEM images, being in good agreement with the assumption that the additional material is present mainly in the form of crystallites.

## 5. Conclusions

To summarize the diffraction experiments, we have observed the formation of a Ga_0.2_In_0.8_As alloy in the presence of Si dopants during the growth of InAs NWs on a GaAs substrate covered with defective Si oxide. This alloy is formed preferentially as parasitic island-type crystallites, growing in between the NWs. The alloy formation can be suppressed when the growth is done on a faultless oxide layer. Nevertheless, the presence of Si adatoms during growth favours the formation of InAs crystallites, which increase in number and volume with increasing Si concentration. On the other hand, we do not find any structural modification of the grown NWs as a function of doping.

The formation of Ga_*x*_In_1−*x*_As alloys has been observed also in other systems, for example during the gold-assisted growth of InAs nanowires on GaAs by metal–organic vapour phase epitaxy (Bauer *et al.*, 2009 ▶). In this system, an initially formed eutectic alloy between deposited Au droplets and Ga, which is dissolved from the GaAs substrate, is enriched by the supplied indium in the growth process, leading to the formation of a Ga_*x*_In_1−*x*_As layer with Ga compositions up to ∼20%. Additionally, the alloy was found in the form of traces, which coalesce during further growth to form eventually a closed layer. Similar traces are also found in the present case, as visible in Fig. 1 ▶(*e*). Although no gold is involved here, we speculate that a eutectic is formed between In and Ga, crystallizing to the observed Ga_*x*_In_1−*x*_As.

While the nucleation of NWs is determined mainly by the number of pinholes in the initial oxide layer, the observed tendency of enhanced crystallite formation with increasing silicon concentration can be ascribed to a change in surface diffusion of the group III materials on the oxide-covered surface, namely a reduced diffusion length. For InAs nanowires grown by metal–organic vapour phase epitaxy, the addition of Si was found to reduce the diffusion length of In on the NW sidewalls, leading to a reduced vertical growth rate of the NWs (Wirths *et al.*, 2011 ▶). The same influence of Si was observed regarding the Ga diffusion length on the sidewalls of MBE-grown GaAs NWs (Dimakis *et al.*, 2012 ▶). In a qualitatively similar way, our findings can be explained assuming that the diffusion length of group III atoms on the oxide layer decreases with increasing Si concentration.

## References

[bb1] Bauer, B., Rudolph, A., Soda, M., Fontcuberta i Morral, A., Zweck, J., Schuh, D. & Reiger, E. (2010). *Nanotechnology*, **21**, 435601. 10.1088/0957-4484/21/43/435601 20876983

[bb2] Bauer, J., Pietsch, U., Davydok, A., Biermanns, A., Grenzer, J., Gottschalch, V. & Wagner, G. (2009). *Appl. Phys. A Mater. Sci. Process.* **96**, 851–859.

[bb3] Blömers, C., Lepsa, M. I., Luysberg, M., Grützmacher, D., Lüth, H. & Schäpers, Th. (2011). *Nano Lett.* **11**, 3550–3556. 10.1021/nl201102a 21848307

[bb4] Davydok, A., Breuer, S., Biermanns, A., Geelhaar, L. & Pietsch, U. (2012). *Nanoscale Res. Lett.* **7**, 109. 10.1186/1556-276X-7-109 PMC3329407 22315928

[bb5] Dimakis, E., Lähnemann, J., Jahn, U., Breuer, S., Hilse, M., Geelhaar, L. & Riechert, H. (2011). *Cryst. Growth Des.* **11**, 4001–4008.

[bb6] Dimakis, E., Ramsteiner, M., Tahraoui, A., Riechert, H. & Geelhaar, L. (2012). *Nano Res.* **5**, 796–804. 10.1021/nl500428v 24678901

[bb7] Fontcuberta i Morral, A., Colombo, C. & Abstreiter, G. (2008). *Appl. Phys. Lett.* **92**, 063112.

[bb8] Kriegner, D., Panse, C., Mandl, B., Dick, K., Keplinger, M., Persson, J., Caroff, P., Ercolani, D., Sorba, L., Bechstedt, F., Stangl, J. & Bauer, G. (2011). *Nano Lett.* **11**, 1483–1489. 10.1021/nl1041512 21434674

[bb9] Krogstrup, P., Popovitz-Biro, R., Johnson, E., Madsen, M. H., Nygård, J. & Shtrikman, H. (2010). *Nano Lett.* **10**, 4475–4482. 10.1021/nl102308k 20932012

[bb10] Li, J., Zhang, Y., To, S., You, L. & Sun, Y. (2011). *ACS Nano*, **8**, 6661–6668. 10.1021/nn202182p 21815637

[bb11] Mandl, B., Stangl, J., Hilner, E., Zakharov, A., Hillerich, K., Dey, A., Samuelson, L., Bauer, G., Deppert, K. & Mikkelsen, A. (2010). *Nano Lett.* **10**, 4443–4448. 10.1021/nl1022699 PMC3028567 20939507

[bb12] Rekaya, S., Bouzaiene, L., Sfaxi, L. & Maaref, H. (2005). *Appl. Phys. A Mater. Sci. Process.* **81**, 79–81.

[bb13] Rieger, T., Heiderich, S., Lenk, S., Lepsa, M. I. & Grützmacher, D. (2012). *J. Cryst. Growth*, **353**, 39–46.

[bb14] Schubert, L., Werner, P., Zakharov, N., Gerth, G., Kolb, F., Long, L., Gösele, U. & Tan, T. (2004). *Appl. Phys. Lett.* **84**, 4968–4970.

[bb15] Stoica, T. & Calarco, R. (2011). *IEEE J. Quantum Electron.* **17**, 4–9.

[bb16] Tang, J., Huo, Z., Brittman, S., Gao, H. & Yang, P. (2011). *Nat. Nanotechnol.* **6**, 568–572. 10.1038/nnano.2011.139 21857684

[bb17] Wagner, R. & Ellis, C. (1964). *Appl. Phys. Lett.* **4**, 89–94.

[bb18] Wallentin, J., Persson, J., Wagner, J., Samuelson, L., Deppert, K. & Borgström, M. (2010). *Nano Lett.* **10**, 974–979. 10.1021/nl903941b 20163125

[bb19] Wei, W., Bao, X., Soci, C., Ding, Z., Wang, Z. & Wang, D. (2009). *Nano Lett.* **9**, 2926–2934. 10.1021/nl901270n 19624100

[bb20] Wirths, S., Weis, K., Winden, A., Sladek, K., Volk, C., Alagha, S., Weirich, T., von der Ahe, M., Hardtdegen, H., Lüth, H., Demarina, N., Grützmacher, D. & Schäpers, Th. (2011). *J. Appl. Phys.* **110**, 053709.

